# Evaluation of the Antibacterial Activity of Gentamicin in Combination with Essential Oils Isolated from Different Cultivars and Morphological Parts of Lavender (*Lavandula angustifolia* Mill.) against Selected Bacterial Strains

**DOI:** 10.3390/molecules28155781

**Published:** 2023-07-31

**Authors:** Michalina Adaszyńska-Skwirzyńska, Sławomir Zych, Mateusz Bucław, Danuta Majewska, Małgorzata Dzięcioł, Danuta Szczerbińska

**Affiliations:** 1 Department of Monogastric Animal Sciences, Faculty of Biotechnology and Animal Husbandry, West Pomeranian University of Technology in Szczecin, Janickiego Str. 29, 71-270 Szczecin, Poland; mateusz.buclaw@zut.edu.pl (M.B.); danuta.majewska@zut.edu.pl (D.M.); danuta.szczerbinska@zut.edu.pl (D.S.); 2 Laboratory of Chromatography and Mass Spectrometry, Faculty of Biotechnology and Animal Husbandry, West Pomeranian University of Technology in Szczecin, Janickiego Str. 29, 71-270 Szczecin, Poland; slawomir.zych@zut.edu.pl; 3 Department of Chemical Organic Technology and Polymeric Materials, Faculty of Chemical Technology and Engineering, West Pomeranian University of Technology in Szczecin, Piastów Ave. 42, 71-065 Szczecin, Poland; malgorzata.dzieciol@zut.edu.pl

**Keywords:** lavender essential oil, gentamicin, antibacterial activity, synergism, *Staphylococcus aureus*, *Pseudomonas aeruginosa*

## Abstract

The aim of the study was to investigate the antibacterial effects of essential oils isolated from different cultivars and morphological parts of lavender (*Lavandula angustifolia* Mill.) in combination with the aminoglycoside antibiotic gentamicin. This in vitro study analyzed the effectiveness of the combinations of gentamicin and lavender essential oils against the following strains: *Staphylococcus aureus* ATCC 25923, *Staphylococcus aureus* MRSA and *Pseudomonas aeruginosa* ATCC 9027. The effect of the combination of lavender oils with gentamicin was tested using the checkerboard method. A synergistic effect against *S. aureus* strain ATCC 25923 was found when gentamicin was combined with lavender essential oils isolated from flowers and leafy stalks (flowers: ‘Blue River’ FICI—0.192; ‘Ellagance Purple’ FICI—0.288; leafy stalks: ‘Blue River’ FICI—0.192; ‘Ellagance Purple’ FICI—0.320). A synergistic effect was also observed for the combination of gentamicin with lavender essential oils from flowers against the resistant strain of *S. aureus* (MRSA) (‘Blue River’ FICI—0,191; ‘Ellagance Purple’ FICI—0.263), as well as for the essential oils from leafy stalks (‘Blue River’ FICI—0.076; ‘Ellagance Purple’ FICI—0.089). No interaction was observed for the combination of studied essential oils with gentamicin against *P. aeruginosa* strain ATCC 9027 (FICI = 1.083–1.300).

## 1. Introduction

Considering the exhausting possibilities of classical therapies in the treatment of bacterial infections, new solutions are constantly being sought. For this purpose, the use of substances of natural origin is increasingly often considered, particularly combination therapies involving concurrent administration of biologically active substances of plant origin, e.g., essential oils, with synthetic antibiotics [1,2,3,4,5,6]. Combination therapy increases the efficacy of antimicrobials agents and prevents strains from acquiring resistance to antibiotics. Importantly, because of the multi-target action of most natural antimicrobial products, there is a negligible risk of generating resistance mechanisms in pathogens [3,7]. There are also other arguments supporting the therapeutic use of natural bioactive substances such as essential oils. Their advantage is demonstrated by a therapeutic effect with a broad spectrum of biological activity [1,5,7]. Lavender (*Lavandula angustifolia* Mill.) essential oils are characterized by antibacterial, antifungal, antioxidant, sedative, analgesic, anti-inflammatory and immunomodulatory properties [4,7,8,9,10,11,12,13,14,15,16,17,18,19,20,21,22,23,24]. Analyses of the antibacterial properties of lavender essential oils have been carried out by a number of researchers. The obtained results indicate that lavender essential oils show a particularly strong action against various Gram-positive bacteria: *Bacillus cereus*, *Bacillus subtilis*, *Listeria monocytogenes*, *Staphylococcus aureus*, *Staphylococcus epidermidis*, *Enterococcus faecalis* or *Streptococcus pyogenes* [4,6,7,11,12,13,14,16,17,18,19,20,21,22]. It has been demonstrated that lavender essential oils also act against Gram-negative bacteria, such as *Campylobacter jejuni*, *Escherichia coli*, *Pseudomonas aeruginosa*, *Salmonella* Typhimurium, *Salmonella* Enteritidis, *Salmonella* Pullorum or *Klebsiella* sp. [4,6,7,11,12,21,23,24]. As a plant with repellent properties, lavender has evolved mechanisms to defend against microbial attacks by producing protective substances, typically originating from secondary metabolism. These mechanisms involve the synthesis of two main types of substances: prohibitins, which are produced to prevent microbial attacks, and phytoalexins, which are synthesized to repair damage of the plant tissues. The compounds with strong bactericidal activity are mostly classified as phytoalexins, which include components of the essential oils. The increase in antimicrobial resistance has a significant impact on public health, global development, and even the world economy [15,25,26]. The incorrect application and overuse of antibiotics without identifying the etiological factor are the driving factors behind increasing drug resistance [25,26]. Therefore, the search for synergism between synthetic antibiotics and antimicrobial agents, including natural substances such as essential oils, present an excellent research direction, being in agreement with ideas surrounding sustainable development. The great advantage is that synergism allows the dose of a drug to be reduced below the threshold of its toxicity [1,4]. Aminoglycoside antibiotics, which include gentamicin, are an important group of chemotherapeutics used to treat severe infections caused by Gram-negative bacteria, both aerobic and anaerobic [26,27]. The use of gentamicin has become a turning point in the treatment of pruritic infections, mainly caused by β-lactam-resistant *Staphylococcus aureus* (MRSA) and *Pseudomonas aeruginosa* strains. Since the 1980s, it has not been possible to obtain new aminoglycosides that offer high efficacy with relatively low toxicity [28]. This has hindered further research on new antibiotics from this group, and current studies mainly focus on mechanisms of drug resistance. Due to the increasing resistance of bacteria and frequent failure of antibiotic therapies, it seems necessary to search for unconventional methods to improve treatment effectiveness. Therefore, the aim of this study was to investigate the effect of the interaction between lavender essential oils obtained from two lavender (*L. angustifolia*) cultivars and their various morphological parts (flowers and leafy stalks) with gentamicin on selected bacterial strains. 

## 2. Results

The chemical composition of lavender essential oils was presented in our previous publication [29]. The main chemical components detected in the essential oils from the flowers of *L. angustifolia* were linalyl acetate (17.8–31.0%) and linalool (28.5–33.0%), while lavandulol acetate, borneol and caryophyllene oxide were present in lower concentrations (<5%). The essential oils from the leafy stalks of *L. angustifolia* were dominated by borneol (13.0–19.7%) and caryophyllene oxide (7.95–10.0%). Chromatographic analysis showed that the relative percentage content of specific compound classes in the flower essential oils was as follows (%): monoterpene hydrocarbons (4.3–4.8), oxygenated monoterpenes (81.6–82.6), sesquiterpene hydrocarbons (6.8–7.7), and oxygenated sesquiterpenes (3.1–3.7). The following compounds were identified in the oils from leafy stalks (%): monoterpene hydrocarbons (7.6–7.7), oxygenated monoterpenes (51.2–53.8), sesquiterpene hydrocarbons (13.0–14.2) and oxygenated sesquiterpenes (8.0–9.0). The essential oils isolated in the study were characterized by varying antimicrobial activity, with *S. aureus* strains being more sensitive to oils from *L. angustifolia*. The growth of bacterial strains was inhibited by the essential oils as follows (MIC % *v*/*v*): *S. aureus* ATCC25922 (0.125–0.5), *S. aureus* MRSA (0.25–1.25), *P. aeruginosa* ATCC 9027 (0.75–2.5). Table 1 presents the results of the interaction between the essential oils isolated from the flowers of *L. angustifolia* in combination with gentamicin, determined using the checkerboard method. Individual interactions of lavender flower essential oils in combination with gentamicin were found be synergistic against *S. aureus* strains (*S. aureus* ATCC 25923: ‘Blue River’ FICI—0.192; ‘Ellagance Purple FICI—0.288; *S. aureus* MRSA: ‘Blue River’ FICI—0.191; ‘Ellagance Purple’ FICI—0.263). A neutral effect was found for the tested combinations against *P. aeruginosa* strain ATCC 9027 (‘Blue River’ FICI—1.083; ‘Ellagance Purple’ FICI—1.25). Similarly, in the case of oils isolated from leafy stalks (Table 2), a synergistic effect was found for *Staphylococcus* strains (*S. aureus* ATCC 25923: ‘Blue River’ FICI—0.192; ‘Ellagance Purple’ FICI—0.320; *S. aureus* MRSA: ‘Blue River’ FICI—0.076; ‘Ellagance Purple’ FICI—0.089). A neutral effect was once again demonstrated for the combination of gentamicin with leafy stalks essential oils against *P. aeruginosa* strain ATCC 9027 (‘Blue River’ FICI—1.166; ‘Ellagance Purple’ FICI—1.300).

## 3. Discussion

Aminoglycoside antibiotics belong to the group of chemotherapeutics that inhibit bacterial protein biosynthesis, and include antibiotics of natural origin, such as gentamicin and tobramycin, as well as semi-synthetic antibiotics like amikacin [26]. Aminoglycosides exert a strong effect on aerobic bacteria, mainly Gram-negative, but also certain Gram-positive bacteria. They exhibit particularly strong antibacterial activity against strains belonging to genera *Escherichia*, *Klebsiella*, *Enterobacter*, *Pseudomonas*, *Hemophilus*, and *Staphylococcus.* Aminoglycosides are substances that contain tri- or tetrasaccharide structures, with streptamine or its derivatives being their common component. The mechanism of action of aminoglycoside antibiotics involves irreversible binding to the 30S ribosomal subunit, which hinders the attachment of aminoacyl-tRNA and causes the misreading of genetic information. This in turn leads to the incorporation of false amino acids, disruption of enzymatic and structural protein synthesis, which initiates irreversible damage to bacterial cell membranes [25,31,32]. Resistance to aminoglycoside antibiotics can develop very rapidly. It involves the formation of enzymes that inactivate aminoglycoside antibiotics. The genetic information of these enzymes is stored in plasmids. Another possible mechanism of microbial resistance to aminoglycoside antibiotics is an alteration in the amino acid sequence of certain ribosomal proteins, which prevents the binding of the antibiotic to the 30S ribosomal subunit. The ability of bacteria to develop antibiotic resistance depends on their adaptability to selective pressure. One of the mechanisms relates to the presence of efflux pumps in bacteria, which allow the antibiotic to be expelled from the cell. Another mechanism involves changes in the composition and function of the cell membrane, which result in its reduced permeability. The next pathway is antibiotic inactivation by extracellular and intracellular enzymes. Bacterial cell adaptation to antibiotic action can involve the reduction of the substrate targeted by the antibiotic or bacterial mutations [31,32]. Generally, two types of antibiotic resistance can be distinguished. The first is natural antibiotic resistance, resulting from the cell’s biology, structural or functional characteristics [32]. The second type is acquired antibiotic resistance. It can occur, for instance, due to chromosomal mutations or through the transfer of genetic material from another bacterium via plasmids [32]. The transfer of genes through plasmids occurs through conjugation, transduction, and transformation, or is facilitated by proteinaceous filaments termed fimbriae or pili [33]. In the transduction process, genes encoding resistance are passed from a resistant bacterial cell to a susceptible one. Antibiotic-sensitive cells can take up genetic material that has been released through autolysis of resistant cells—a process known as transformation [33,34]. The antibiotic resistance profile of *Staphylococcus* strains continues to evolve, contributing to the spread of new bacterial clones [35,36,37]. 

Most of the essential oils contain one main chemical component that is responsible for their biological and pharmacological properties [7,38,39]. The chemical composition of essential oil may vary significantly creating their unique chemotypes within plant species with the same morphological features. Additionally, the composition depends on many factors, such as the country of origin, the part of the plant and the production method [40]. For example, cinnamon essential oil contains 50–75% of trans-cinnamaldehyde when extracted from the bark of the true cinnamon tree (*Cinnamomum zeylanicum*). However, the oil produced from the leaves of this tree contains 50–75% of eugenol and only a trace amount of cinnamaldehyde, making it similar in composition, activity, and scent to clove essential oil [41]. Camphor is derived from the wood, bark, and leaves of *Cinnamomum camphora* (as “camphor brown” oil). However, the first distillation fraction is referred to as “camphor white” mainly containing 1,8-cineole (30–60%; similar to eucalyptus oil) [42]. In contrast, essential oil distilled from *C. camphora* var. *linaloolifera* (also known as Ho wood) typically contains more than 90% of linalool [43]. Each of these main components has demonstrated a broad range of highly antimicrobial activities against different pathogens [40]. Information available in the literature indicates that linalool is the chemical compound responsible for the antibacterial activity of the essential oil from *L. angustifolia*. However, it should be emphasized that essential oils derived from leafy stalks are characterized by low content of linalool, yet they still exhibit antibacterial activity. The overall concept of antimicrobial synergy is based on the principle that the combination of two or more antimicrobial agents (even in lower amounts) may enhance their efficacy. This is manifested, for example, due to their hydrophobicity, by reaction with lipids present in the bacterial cell membrane (e.g., destroying cell structures or increasing the permeability of the main component), a reduction in the required dose of the active substance or reduced antimicrobial resistance [44]. The biosynthesis of secondary metabolites and their proportion in essential oils vary due to environmental factors, such as seasonal variation, light availability, vegetation stage, presence of additional commensal microorganisms as well as nature of soil (e.g., pH) [42]. Moreover, Gram-positive bacteria seem to be much more susceptible to essential oil than Gram-negative [4]. All tested lavender essential oils exhibited more effective antibacterial activity against *S. aureus* strains (MIC % *v*/*v*: 0.125–1.25) compared to *P. aeruginosa* strains (MIC % *v*/*v*: 0.75–2.50). This suggests that lavender oils may show weaker activity against Gram-negative bacteria in general, particularly the genus *Pseudomonas*, which naturally occurs only in soil and water. *Pseudomonas* has developed a range of cellular defense mechanisms through adaptation to adverse environmental conditions, which may explain the observed lower susceptibility to lavender essential oils compared to *S. aureus*. Different antibacterial activity of essential oils can be possibly related to its chemical composition and content of some other compounds with synergistic activity. Available literature indicates the large variability in antimicrobial properties of essential oils isolated from *L. angustifolia* flowers. Strong antimicrobial activity against the reference strains of *S. aureus* CECT 4459 with an MIC level of 0.0025% *w*/*v* was demonstrated by Djenane et al. [45], while weak activity was reported by Thosar et al. with an MIC value of 0.003% *w*/*v* against *S. aureus* ATCC 25923 [46]. In turn, according to De Martino et al. *L. angustifolia* essential oil did not show any activity against strains of *S. aureus* and *P. aeruginosa* [12]. In studies performed by de Rapper et al., the value of lavender essential oil MIC against *S. aureus* MRSA and *P. aeruginosa* ATCC 11093 was 0.2% *w*/*v*, regardless of whether *Staphylococcus*, MRSA or gentamicin-resistant MRSA was tested (strain analogous to that tested in our study) [47]. 

In our studies, after analyzing individual combinations of gentamicin with lavender essential oils, it was observed that their effects on tested microorganisms varied. Some combinations indicated synergism (*S. aureus* ATCC 25923 FICI: 0.192–0.32; *S. aureus* MRSA FICI: 0.076–0.263), while others showed lack of interaction (*P. aeruginosa* ATCC 9027 FICI: 1.08–1.30). It should be emphasized that an antagonistic interaction was not recorded in any case. No data were found in the literature on the effect of essential oils from different cultivars and morphological parts of lavender in combination with gentamicin. The conducted research indicates that their biological activity slightly varied among two studied lavender cultivars and was also affected by the part of the plant used for the isolation of essential oil.

Importantly, a synergistic interaction between gentamicin and lavender essential oils was obtained against the resistant isolate (*S. aureus* MRSA), which was included in the list of pathogens urgently requiring development of new antibiotics by the World Health Organization (WHO) in 2017 [48]. A very interesting observation is that essential oils isolated from the leafy stalks of both studied lavender cultivars are very effective enhancers of gentamicin activity against this resistant strain. Upon comparing the obtained FICI values, it can be noticed that the ability to enhance the gentamicin activity was significantly higher in the case of both ‘Blue River’ and ‘Ellagance Purple’ cultivars in comparison to essential oils obtained from their flowers. These results indicate that the essential oil from the leafy stalks of lavender, despite the lower content of linalool, can also be a valuable natural bioactive product with potential practical application. It also confirms that the antimicrobial activity of lavender essential oils is the result of more than linalool content alone, and further research for the possible explanation of this fact is required. For this purpose, the biological activity of other major and minor components of lavender essential oils should be tested in analogous conditions to identify the remaining active compounds.

Some authors have shown synergistic effects with gentamicin for other essential oils against Gram-positive and Gram-negative bacteria [1,44,49,50,51]. This was confirmed in our previous study on commercial lavender essential oil [7] and the currently studied oils isolated from different cultivars and morphological parts of lavender, whose combinations with gentamicin have not yet been examined. The obtained results demonstrate synergy between the tested essential oils and gentamicin and are thus very promising. Further research is necessary to confirm the effectiveness of these combinations through in vivo studies.

## 4. Materials and Methods

### 4.1. Isolation of Essential Oils

Plant raw material comprised dried flowers and leafy stalks of two cultivars of *Lavandula angustifolia*: ‘Blue River’ and ‘Ellagance Purple’. Directly before isolation of essential oils, plant material was pounded in a mortar and weighed sample (20.00 g) was placed in a 1000 mL round-bottomed flask, immersed in 400 mL distilled water, and connected to Deryng apparatus. Hydrodistillation process was performed for 3 h and the obtained oils were dried over anhydrous sodium sulfate. For this purpose, 1.0 mL of methylene chloride and desiccant were added to the vial with essential oil. After storage in the refrigerator for 24 h, the drying agent was removed by filtration and the solvent was evaporated to obtain a pure essential oil. All isolated essential oils were analyzed using gas chromatography with mass selective detector (GC-MS), what was described previously [29]. (See Supplementary Materials for details). 

### 4.2. Microdilution Checkerboard Method

Two bacterial strains of the American Type Culture Collection (*S. aureus* ATCC 25923, *P. aeruginosa* ATCC 9027, KwikStik™, Microbiologics, St. Cloud, MS, USA) and one isolate from purulent skin lesions in a horse (*S. aureus* MRSA—bacteria from the culture collection of the Labo-Wet Laboratory, Szczecin, Poland) were used for the study. *S. aureus* and *P. aeruginosa* are versatile bacterial pathogens and common etiological agents in polymicrobial infections. Both *S. aureus* and *P. aeruginosa* exhibit intrinsic and acquired antibiotic resistance, making infections by these pathogens increasingly difficult to treat. Microbial communities containing both pathogens are shaped by interactions ranging from parasitic to mutualistic, with the net impact of these interactions in many cases resulting in enhanced virulence. Moreover, synergism between *S. aureus* and *P. aeruginosa* has been observed in multiple models of infection, including wounds and chronic lung infection. The disc-diffusion method was used to determine the preliminary classification of the tested strains for susceptibility to gentamicin (commercial disc 10 μg, OXOID, Argenta, Poznan, Poland). Interpretive criteria of zone diameter (mm) were used according to CLSI VET01S 5th edition (*Staphylococci* and *P. aeruginosa* isolated from humans and most animal species: ≤12 (R) resistant, 13–14 (I) intermediate, ≥15 (S) susceptible) [52]. Gentamicin (256 mg/mL; Oxoid Limited, Hampshire, England) diluted in pure deionised water was used for microdilution testing as the active agent, and isolated lavender oils. Dimethylsulfoxide—DMSO (POCH, Gliwice, Poland) was used as an organic solvent to dilute only the oil. In order to determine the susceptibility of the tested microorganisms to the analysed antimicrobial agents, the determination of minimum inhibitory concentration (MIC). All identifications were made on individually packed, sterile, divided, 96-well polystyrene titration plates with flat bottoms. Again, interpretive criteria of MIC (µg/mL) were used according to CLSI VET01S 5th edition (≥16 (R), 8 (I), ≤4 (S) for *Staphylococci* and *P. aeruginosa* isolated from humans and most animals’ species [52]. The Clinical and Laboratory Standard Institute (CLSI) recommendations were followed [52,53]. Each test was repeated three times. The medium was used as a ready-to-use cation-adjusted Mueller–Hinton broth (CAMHB) (Graso, Starogard Gdański, Poland). The final volume of broth in each well was 100 µL. For gentamicin, a series of two-fold dilutions were made in the range of 0.002 ÷ 256 mg/mL, and for essential oils, a concentration gradient was prepared in the range of was 0.008 ÷ 5% *v*/*v*. To avoid the toxicity of DMSO, the final concentration of DMSO in the well did not exceed 5% *v*/*v* for *P. aeruginosa* and 10% *v*/*v* for both *Staphylococci*. Colonies of each strain were selected from 18- to 24-h nonselective blood agar plates. The inoculum of 1–2 × 10^8^ CFU/mL, corresponding to 0.5 McFarland standard measured in a DEN-1 densitometer (BioSan, Józefów, Poland) were employed in the tests (according to CLSI, the final concentration in each well was approximately 5 × 10^5^ CFU/mL). The microtiter plate was incubated in an incubator at +35.0 ± 2 °C for 18 ± 2 h. Due to natural clouding of the medium at high concentrations of the essential oil and DMSO, the result was read macroscopically, and taken into account accordingly. Both antimicrobial agents were considered inefficient when clouding characteristic only for bacteria growth was present. The study of the effect of gentamicin in combination with lavender oils was carried out using the checkerboard method, according to the methodology described in the previous article [7]. MIC data of the gentamicin and lavender essential oils were converted into Fractional Inhibitory Concentration (FIC) and defined as the antimicrobial concentration in an inhibitory concentration with a second compound to the concentration of the antimicrobial by itself [30]. In the combination assays, the checkerboard procedure described by Rosato et al. [49] was followed to evaluate the synergistic action of the lavender essential oils with gentamicin. In our experimental protocol, the substance combinations were analysed by calculating the FIC index (FICI) using the following formulas:

(1) $$\mathrm{FIC}\;\mathrm{of}\;\mathrm{essential}\;\mathrm{oil}=\frac{\mathrm{MIC}\;\mathrm{value}\;\mathrm{of}\;\mathrm{essential}\;\mathrm{oil}\;\mathrm{combined}\;\mathrm{with}\;\mathrm{gentamicin}}{\mathrm{MIC}\;\mathrm{value}\;\mathrm{of}\;\mathrm{essential}\;\mathrm{oil}\;\mathrm{alone}}\;$$

(2) $$\mathrm{FIC}\;\mathrm{of}\;\mathrm{gentamicin}=\frac{\mathrm{MIC}\;\mathrm{value}\;\mathrm{of}\;\mathrm{gentamicin}\;\mathrm{combined}\;\mathrm{with}\;\mathrm{essential}\;\mathrm{oil}\;}{\mathrm{MIC}\;\mathrm{value}\;\mathrm{of}\;\mathrm{gentamicin}\;\mathrm{alone}}\;\;\;$$

(3) FICI=FIC value of essential oil+FIC value of gentamicin 

Generally, the FICI value was interpreted as: synergistic when ≤0.5; additive when >0.5 and ≤1; noninteractive (>1 but ≤4) and antagonistic when >4 [30]. 

## 5. Conclusions

New therapeutic solutions are currently being sought as the problem of drug resistance continues to increase. One of the strategies includes searching for combined treatment options using natural biologically active products with known drugs. For the tested combinations of lavender essential oils with gentamicin against *P. aeruginosa* ATCC 9027 strain, no interaction was found. However, the conducted analyses demonstrated the possibility of using lavender essential oils to enhance the antibacterial activity of gentamicin against *S. aureus* ATCC 25923 and MRSA strains. Despite some differences between the essential oils obtained from the two tested lavender cultivars (‘Blue River’ and ‘Ellagance Purple’), their flowers and leafy stalks, synergistic effects with gentamicin were found in all cases. The obtained results are very promising and important, especially in the context of searching for new possibilities of treating infections in both humans and animals regarding antibiotic-resistant strains. Further, the presented findings encourage and justify further research to verify the results in vivo, aimed at developing antibiotic formulations with biologically active natural substances, characterized by increased effectiveness and lower toxicity.

## Figures and Tables

**Table 1:** Activity of lavender essential oils from flowers in combination with gentamicin against selected bacterial strains.

Antibacterial Agent	Blue River				Ellagance Purple				Type of Interaction
	MIC_0_	MIC_c_	FIC	FICI	MIC_0_	MIC_c_	FIC	FICI	
*Staphylococcus aureus* ATCC 25923									
Essential oil (% *v*/*v*)	0.125	0.016	0.128	0.192	0.250	0.008	0.032	0.288	synergistic
Gentamicin (µg/mL)	0.250	0.016	0.064		0.125	0.032	0.256		
*Staphylococcus aureus* MRSA									
Essential oil (% *v*/*v*)	0.250	0.032	0.128	0.191	1.250	0.016	0.013	0.263	synergistic
Gentamicin (µg/mL)	16.0	1.0	0.063		16.0	4.0	0.250		
*Pseudomonas aeruginosa* ATCC 9027									
Essential oil (% *v*/*v*)	0.750	0.625	0.833	1.083	2.0	1.500	0.750	1.250	no interaction
Gentamicin (µg/mL)	2.0	0.500	0.250		2.0	1.0	0.5		

Explanations: MIC—minimal inhibitory concentration; FIC—fractional inhibitory concentration; FICI—fractional inhibitory concentration index; MIC_0_ = MIC of an individual sample alone; MIC_c_ = MIC of an individual sample of the most effective combination; FIC of essential oil = MIC of essential oil in combination with gentamicin/MIC of essential oil alone; FIC of gentamicin = MIC of gentamicin in combination with essential oil /MIC of gentamicin alone; FICI = FIC of essential oil + FIC of gentamicin. Interpretation: FICI ≤ 0.5, synergistic; FICI > 0.5–1.0, additive; FICI > 1.0–4.0, no interaction; FICI > 4.0, antagonistic [30].

**Table 2:** Activity of lavender essential oils from leafy stalks in combination with gentamicin against selected bacterial strains.

Antibacterial Agent	Blue River				Ellagance Purple				Type of Interaction
	MIC_0_	MIC_c_	FIC	FICI	MIC_0_	MIC_c_	FIC	FICI	
*Staphylococcus aureus* ATCC 25923									
Essential oil (% *v*/*v*)	0.500	0.032	0.164	0.192	0.250	0.016	0.064	0.320	synergistic
Gentamicin (µg/mL)	0.125	0.016	0.128		0.125	0.032	0.256		
*Staphylococcus aureus* MRSA									
Essential oil (% *v*/*v*)	1.250	0.016	0.013	0.076	1.250	0.032	0.026	0.089	synergistic
Gentamicin (µg/mL)	32.0	2.0	0.063		32.0	2.0	0.063		
*Pseudomonas aeruginosa* ATCC 9027									
Essential oil (% *v*/*v*)	1.50	1.0	0.666	1.166	2.5	2.0	0.80	1.300	no interaction
Gentamicin (µg/mL)	2.0	1.0	0.500		2.0	1.0	0.50		

Explanations: MIC—minimal inhibitory concentration; FIC—fractional inhibitory concentration; FICI—fractional inhibitory concentration index; MIC_0_ = MIC of an individual sample alone; MIC_c_ = MIC of an individual sample of the most effective combination; FIC of essential oil = MIC of essential oil in combination with gentamicin/MIC of essential oil alone; FIC of gentamicin = MIC of gentamicin in combination with essential oil/MIC of gentamicin alone; FICI = FIC of essential oil + FIC of gentamicin. Interpretation: FICI ≤ 0.5, synergistic; FICI > 0.5–1.0, additive; FICI > 1.0–4.0, no interaction; FICI > 4.0, antagonistic [30].

## Data Availability

Not applicable.

## Supplementary Materials

The following supporting information can be downloaded at: https://www.mdpi.com/article/10.3390/molecules28155781/s1, GC-MS data: Table S1. Retention parameters of compounds identified in essential oils from flowers and leafy stalks of *Lavandula angustifolia* cultivars: ‘Blue River’ and ‘Ellagance Purple’, obtained by GC-MS method; Figure S1. Chromatogram of essential oil from flowers of ‘Blue River’ cultivar of *Lavandula angustifolia*; Figure S2. Chromatogram of essential oil from flowers of ‘Ellagance Purple’ cultivar of *Lavandula angustifolia*; Figure S3. Chromatogram of essential oil from leafy stalks of ‘Blue River’ cultivar of *Lavandula angustifolia*; Figure S4. Chromatogram of essential oil from leafy stalks of ‘Ellagance Purple’ cultivar of *Lavandula angustifolia*; Figure S5. Mass spectrum of eucalyptol present in *Lavandula angustifolia* essential oils, compared with eucalyptol standard mass spectrum from NIST 02 library; Figure S6. Mass spectrum of linalool present in *Lavandula angustifolia* essential oils, compared with linalool standard mass spectrum from NIST 02 library; Figure S7. Mass spectrum of borneol present in *Lavandula angustifolia* essential oils, compared with borneol standard mass spectrum from NIST 02 library; Figure S8. Mass spectrum of linalool acetate present in *Lavandula angustifolia* essential oils, compared with linalool acetate standard mass spectrum from NIST 02 library; Figure S9. Mass spectrum of lavandulol acetate present in *Lavandula angustifolia* essential oils, compared with lavandulol acetate standard mass spectrum from NIST 02 library; Figure S10. Mass spectrum of caryophyllene present in *Lavandula angustifolia* essential oils, compared with caryophyllene standard mass spectrum from NIST 02 library; Figure S11. Mass spectrum of caryophyllene oxide present in *Lavandula angustifolia* essential oils, compared with caryophyllene oxide standard mass spectrum from NIST 02 library; Figure S12. Chromatogram of linalool standard; Figure S13. Mass spectrum of linalool, compared with linalool standard mass spectrum from NIST 02 library.

## Sample Availability

Samples of essential oils are available from the authors.
