# Supplementary material for: Evaluation of the Antibacterial Activity of Gentamicin in Combination with Essential Oils Isolated from Different Cultivars and Morphological Parts of Lavender (Lavandula angustifolia Mill.) against Selected Bacterial Strains
Source: Molecules. 2023 Jul 31;28(15):5781. doi: 10.3390/molecules28155781 (PMC10421019; doi:10.3390/molecules28155781)
Supplement: Supplementary file 1 [file molecules-28-05781-s001.zip › molecules-2500446-supplementary.pdf]

PART I - Retention parameters of *Lavandula angustifolia* essential oils components

Table S1. Retention parameters of compounds identified in essential oils from flowers and leafy stalks of *Lavandula angustifolia* cultivars: 'Blue River' and 'Ellagance Purple', obtained by GC-MS method

Compound	Retention time RT [min]	Retention index[1] RI	Reference retention index[2] RI_{Ref}
α-Pinene	4.89	934	936
Camphene	5.23	947	950
β-Pinene	5.94	976	977
3-Octanone	6.39	980	980
β-Myrcene	6.77	989	989
Hexyl acetate	6.81	1009	1010
Car-3-ene	6.84	1010	1011
p-Cymene	7.25	1021	1024
Limonene	7.31	1028	1029
Eucalyptol	7.38	1031	1031
trans-β-Ocimene	7.76	1036	1038
cis-β-Ocimene	8.16	1045	1048
cis-Linalool oxide	8.64	1074	1075
trans-Linalool oxide	9.09	1084	1085
Linalool	9.50	1102	1099
1-Octen-3-ol acetate	9.84	1110	1110
Pinocarveol	10.61	1139	1140
Camphor	10.67	1142	1144
Borneol	11.30	1165	1166
4-Terpineol	11.65	1177	1177
p-Cymene-8-ol	11.92	1184	1184
Cryptone	11.97	1187	1187
α-Terpineol	12.10	1190	1191
Myrtenal	12.20	1194	1194
Myrtenol	12.26	1198	1196
Eucarvone	12.84	1201	1199
Chrysanthenone	13.00	1210	-
Carveol	13.46	1225	1224
Nerol	13.49	1229	1230
Cuminal	13.74	1237	1236
Neral	13.75	1239	1240
Carvon	13.79	1244	1242
Linalool acetate	13.85	1256	1256
Phellandral	14.57	1276	1277
Bornyl acetate	14.62	1279	1282
Lavandulol acetate	14.81	1288	1291
Cuminol	15.65	1292	1295
Nerol acetate	16.76	1360	1364
Geraniol acetate	17.25	1378	1380
Caryophyllene	18.06	1417	1421
α-Santalene	18.08	1420	1422
α-Bergamotene	18.50	1430	1434
β-Farnesene	19.07	1453	1456
Germacrene D	19.68	1478	1480
δ-Cadinene	20.44	1519	1522
Caryophyllene oxide	22.08	1579	1577
epi-Bicyclosesquiphellandrene	23.42	1640	1642

[1] Linear retention index determined experimentally in relation to n-alkanes (C_7-C_{30}) on a HP5-MSI column;
[2] Reference linear retention index from the literature: Babushok, V.I., Linstrom, P.J., Zenkevich, I.G. (2011). *Retention indices for frequently reported compounds of plant essential oils.* J. Phys. Chem. Ref. Data. 40(4): 1-47.

PART II - GC-MS data of *Lavandula angustifolia* essential oils

Total Ion Chromatograms obtained by GC-MS method:

Figure S1. Chromatogram of essential oil from flowers of ‘Blue River’ cultivar of *Lavandula angustifolia*

Figure S2. Chromatogram of essential oil from flowers of ‘Ellagance Purple’ cultivar of *Lavandula angustifolia*

Figure S3. Chromatogram of essential oil from leafy stalks of ‘Blue River’ cultivar of *Lavandula angustifolia*

Figure S4. Chromatogram of essential oil from leafy stalks of ‘Ellagance Purple’ cultivar of *Lavandula angustifolia*

Mass spectra of the main components of essential oils:

Figure S5. Mass spectrum of eucalyptol present in *Lavandula angustifolia* essential oils, compared with eucalyptol standard mass spectrum from NIST 02 library

Figure S6. Mass spectrum of linalool present in *Lavandula angustifolia* essential oils, compared with linalool standard mass spectrum from NIST 02 library

Figure S7. Mass spectrum of borneol present in *Lavandula angustifolia* essential oils, compared with borneol standard mass spectrum from NIST 02 library

Figure S8. Mass spectrum of linalool acetate present in *Lavandula angustifolia* essential oils, compared with linalool acetate standard mass spectrum from NIST 02 library

Figure S9. Mass spectrum of lavandulol acetate present in *Lavandula angustifolia* essential oils, compared with lavandulol acetate standard mass spectrum from NIST 02 library

Figure S10. Mass spectrum of caryophyllene present in *Lavandula angustifolia* essential oils, compared with caryophyllene standard mass spectrum from NIST 02 library

Figure S11. Mass spectrum of caryophyllene oxide present in *Lavandula angustifolia* essential oils, compared with caryophyllene oxide standard mass spectrum from NIST 02 library

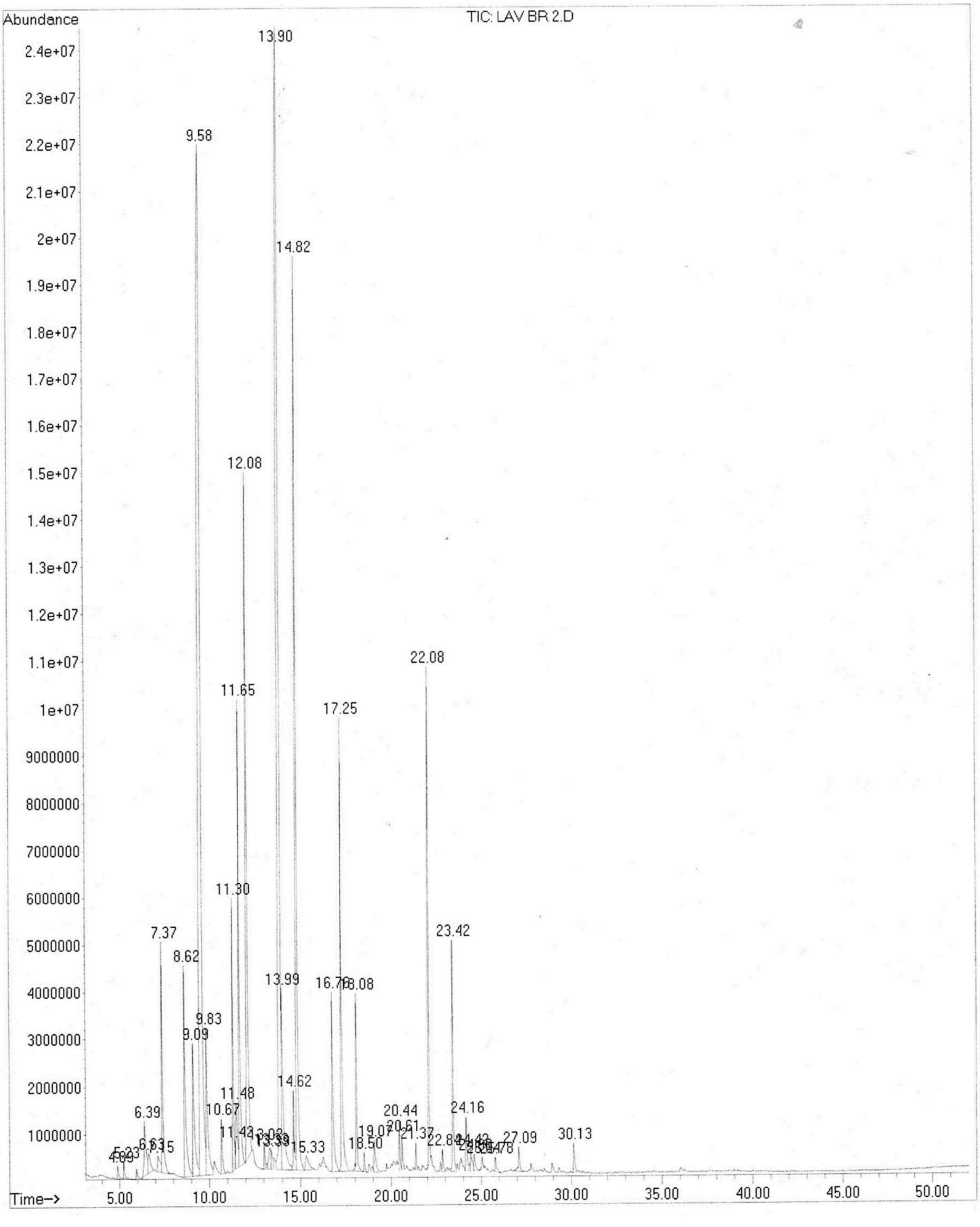

Figure S1. Chromatogram of essential oil from flowers of 'Blue River' cultivar of *Lavandula angustifolia*

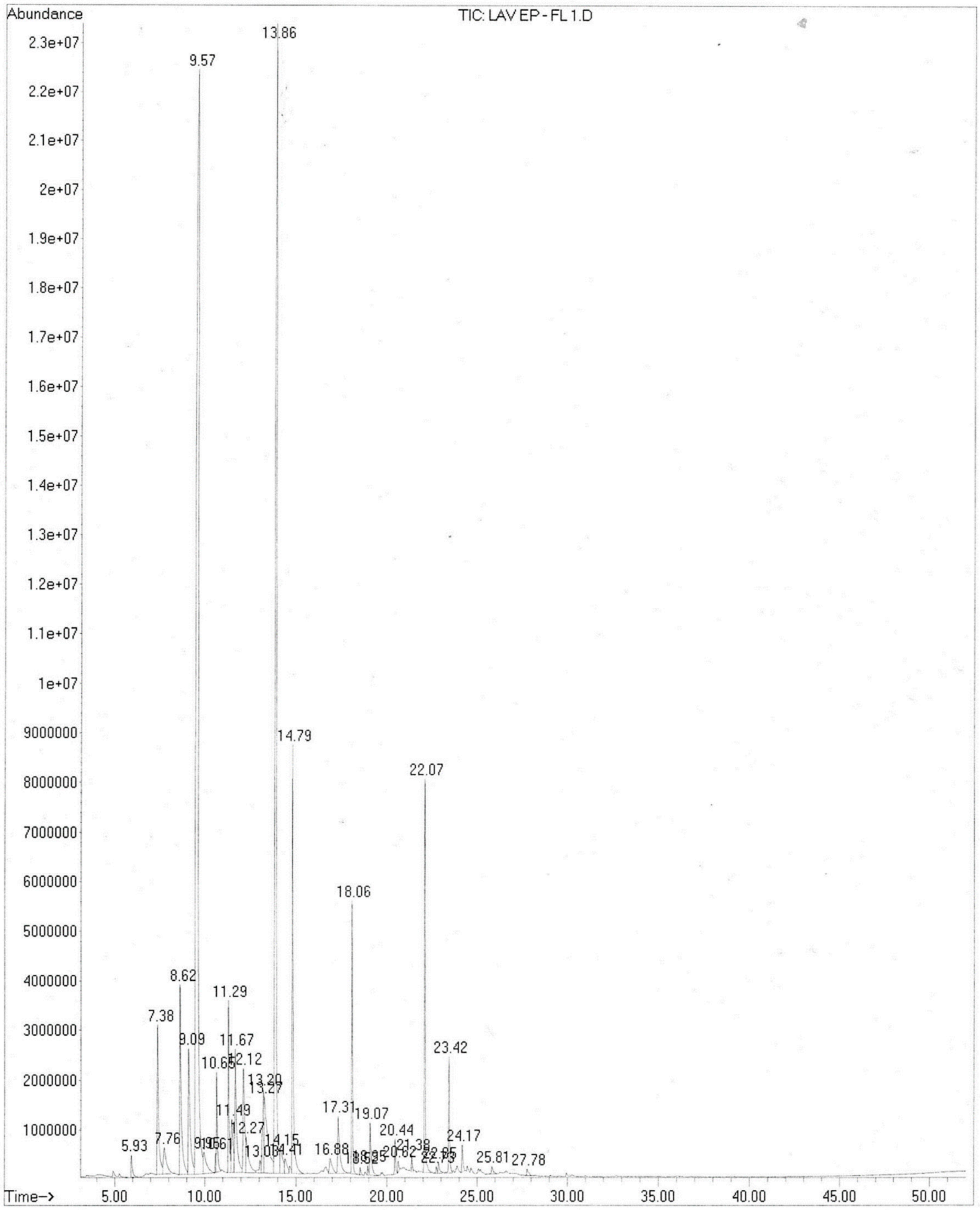

Figure S2. Chromatogram of essential oil from flowers of 'Ellagance Purple' cultivar of *Lavandula angustifolia*

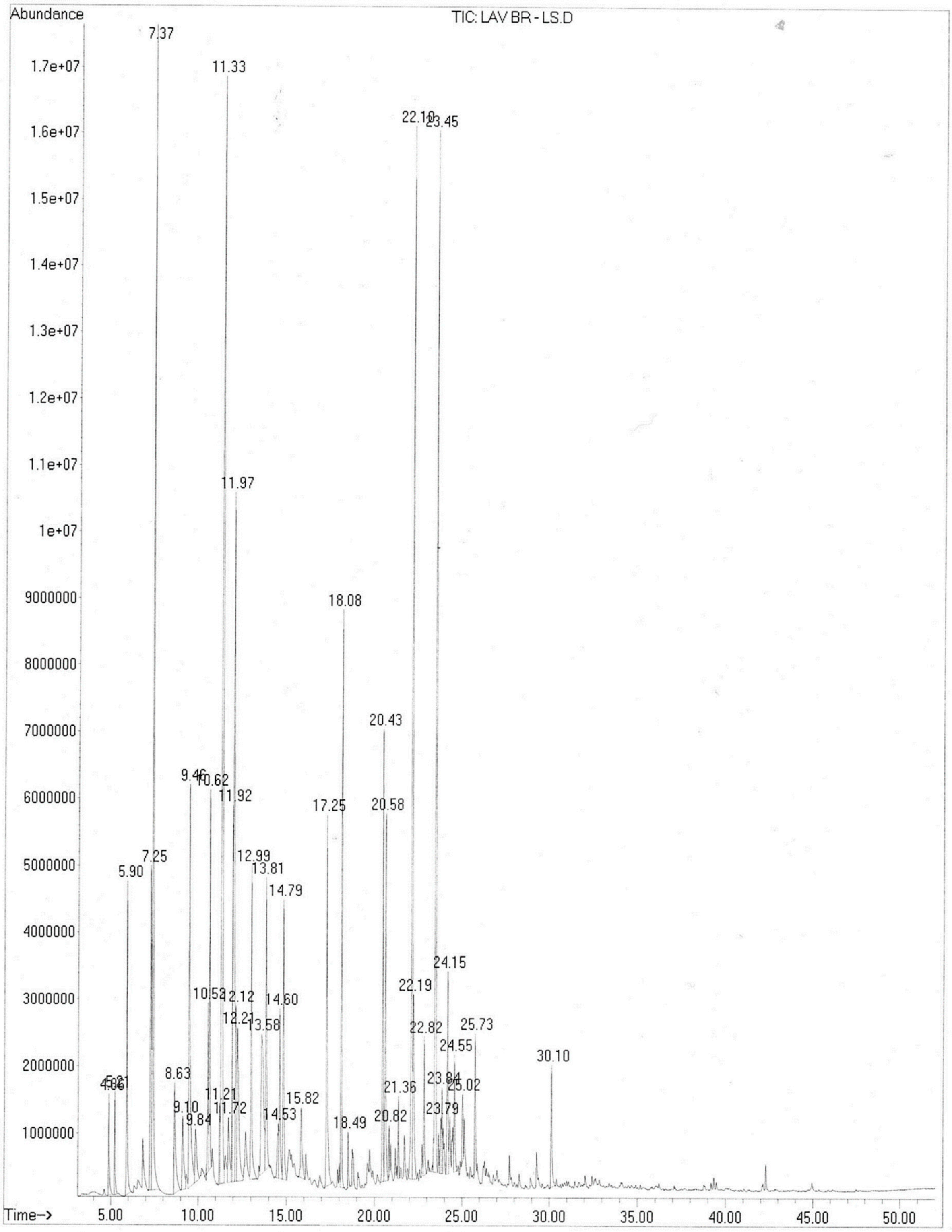

Figure S3. Chromatogram of essential oil from leafy stalks of 'Blue River' cultivar of *Lavandula angustifolia*

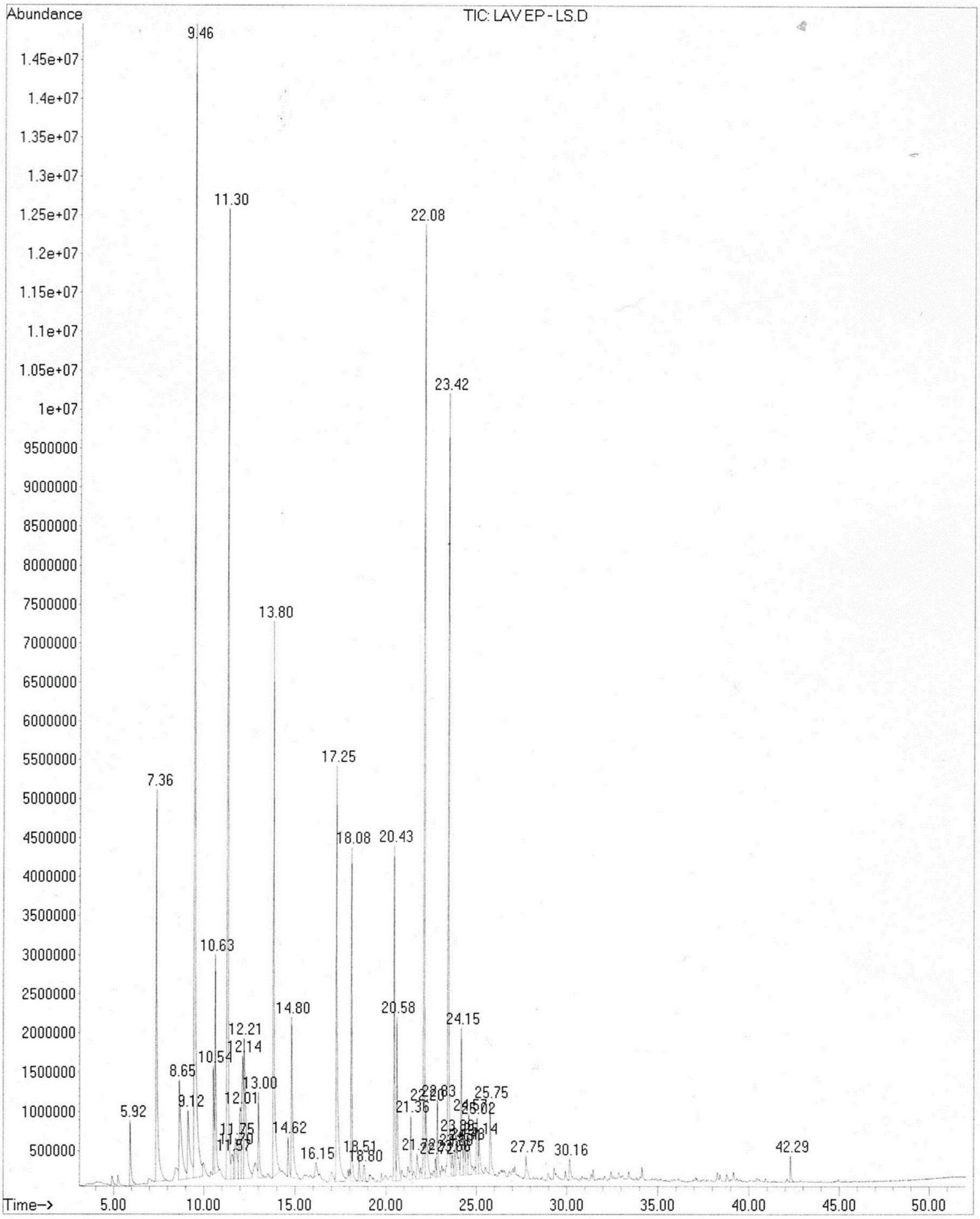

Figure S4. Chromatogram of essential oil from leafy stalks of 'Ellagance Purple' cultivar of *Lavandula angustifolia*

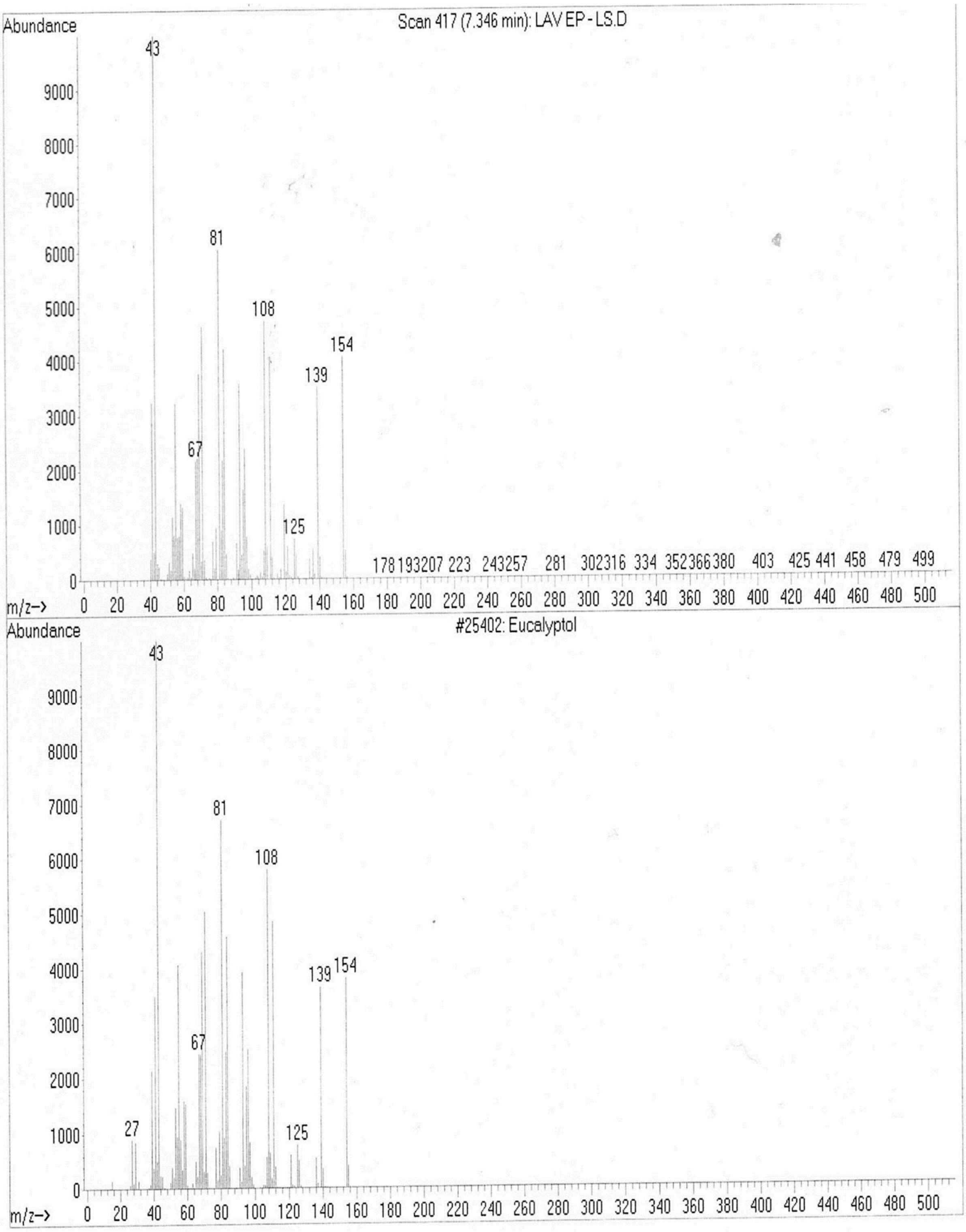

Figure S5. Mass spectrum of eucalyptol present in *Lavandula angustifolia* essential oils, compared with eucalyptol standard mass spectrum from NIST 02 library

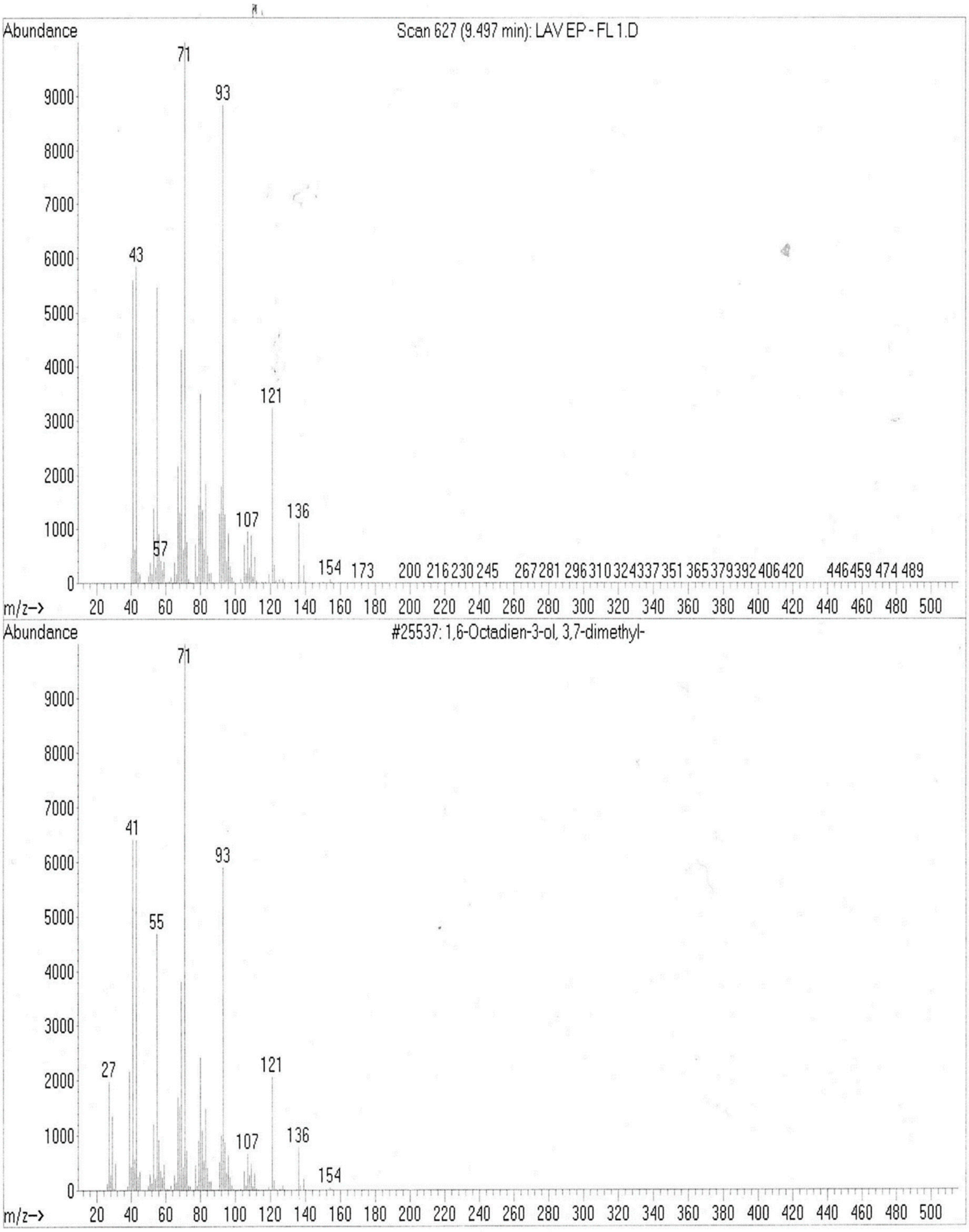

Figure S6. Mass spectrum of linalool present in *Lavandula angustifolia* essential oils, compared with linalool standard mass spectrum from NIST 02 library

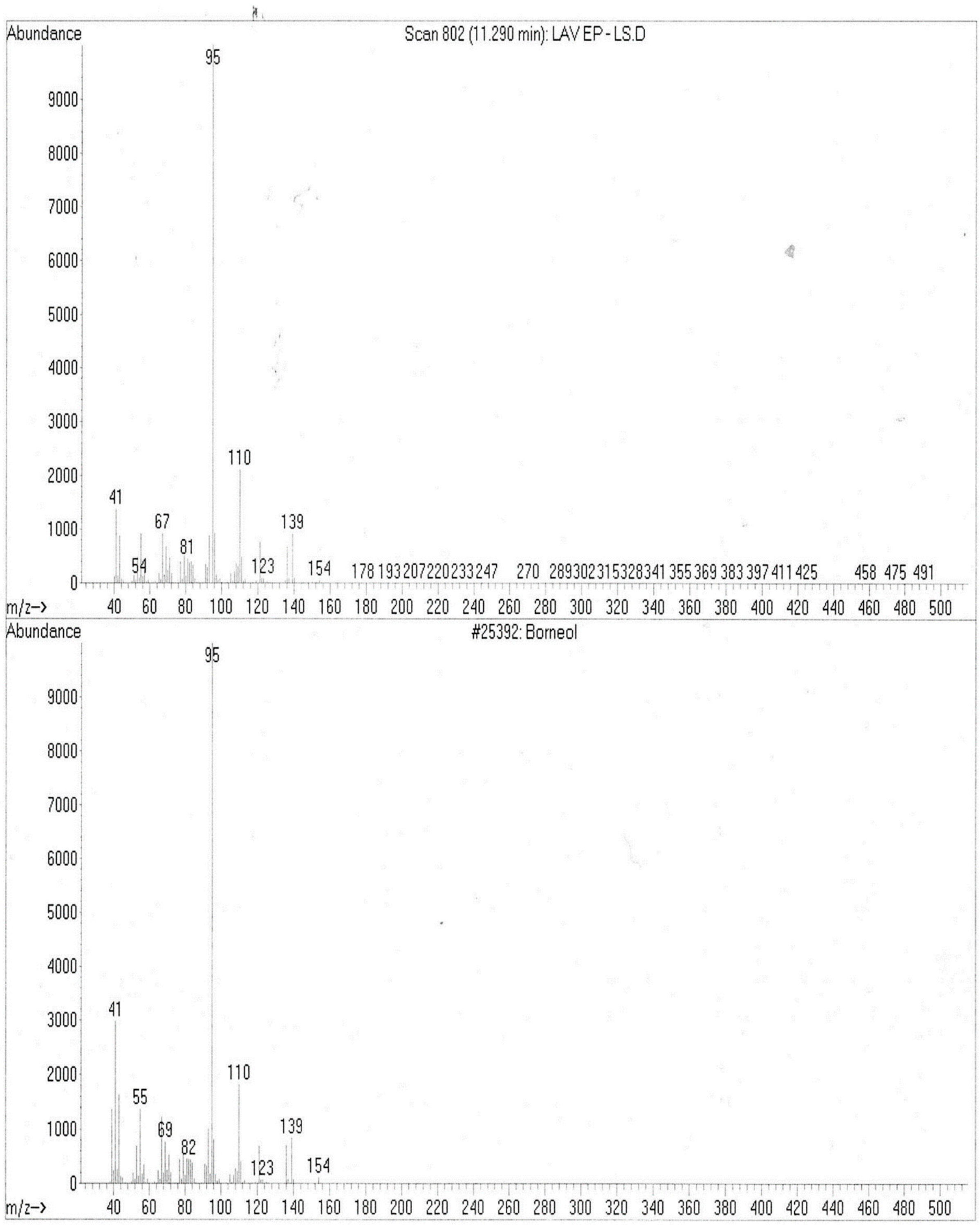

Figure S7. Mass spectrum of borneol present in *Lavandula angustifolia* essential oils, compared with borneol standard mass spectrum from NIST 02 library

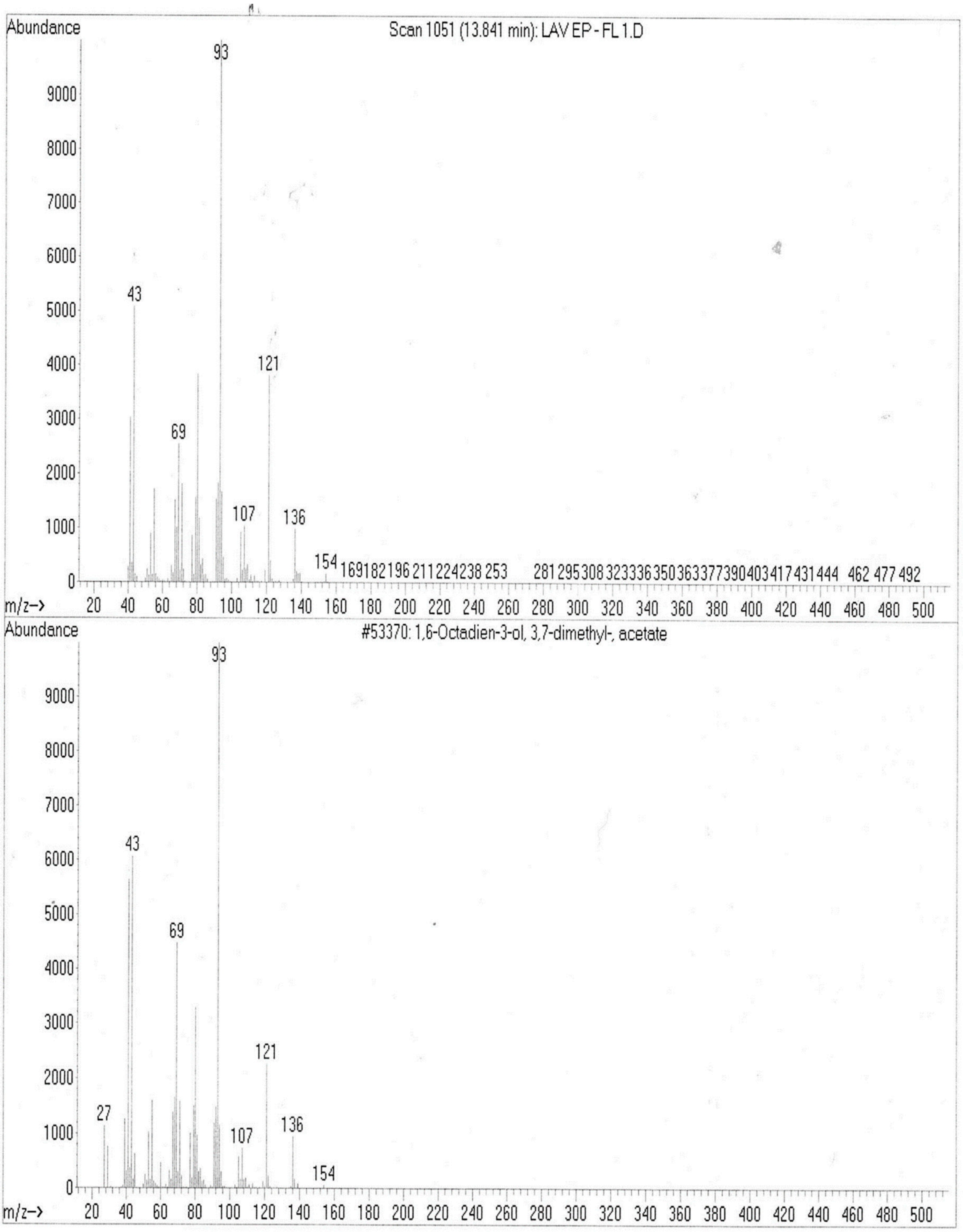

Figure S8. Mass spectrum of linalool acetate present in *Lavandula angustifolia* essential oils, compared with linalool acetate standard mass spectrum from NIST 02 library

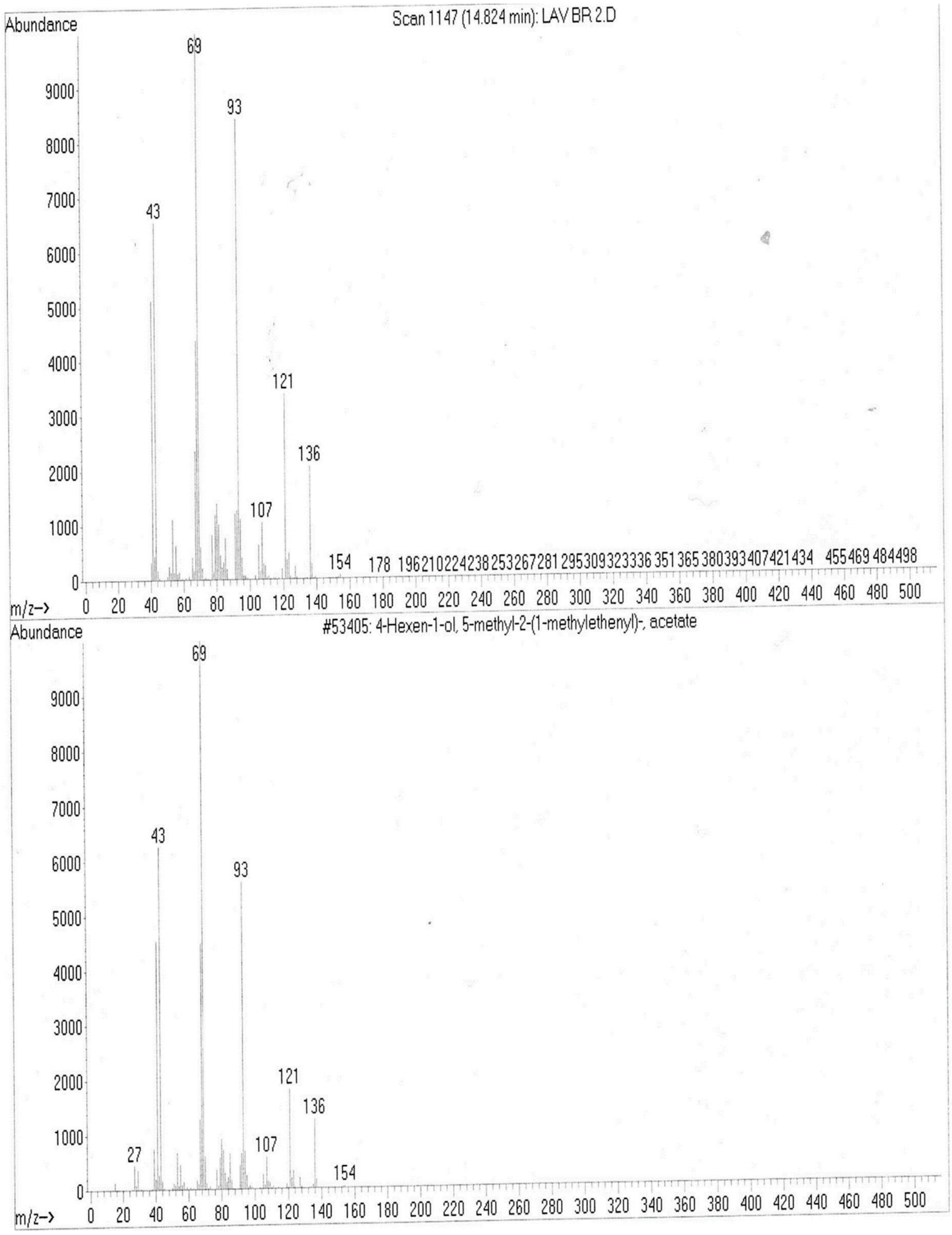

Figure S9. Mass spectrum of lavandulol acetate present in *Lavandula angustifolia* essential oils, compared with lavandulol acetate standard mass spectrum from NIST 02 library

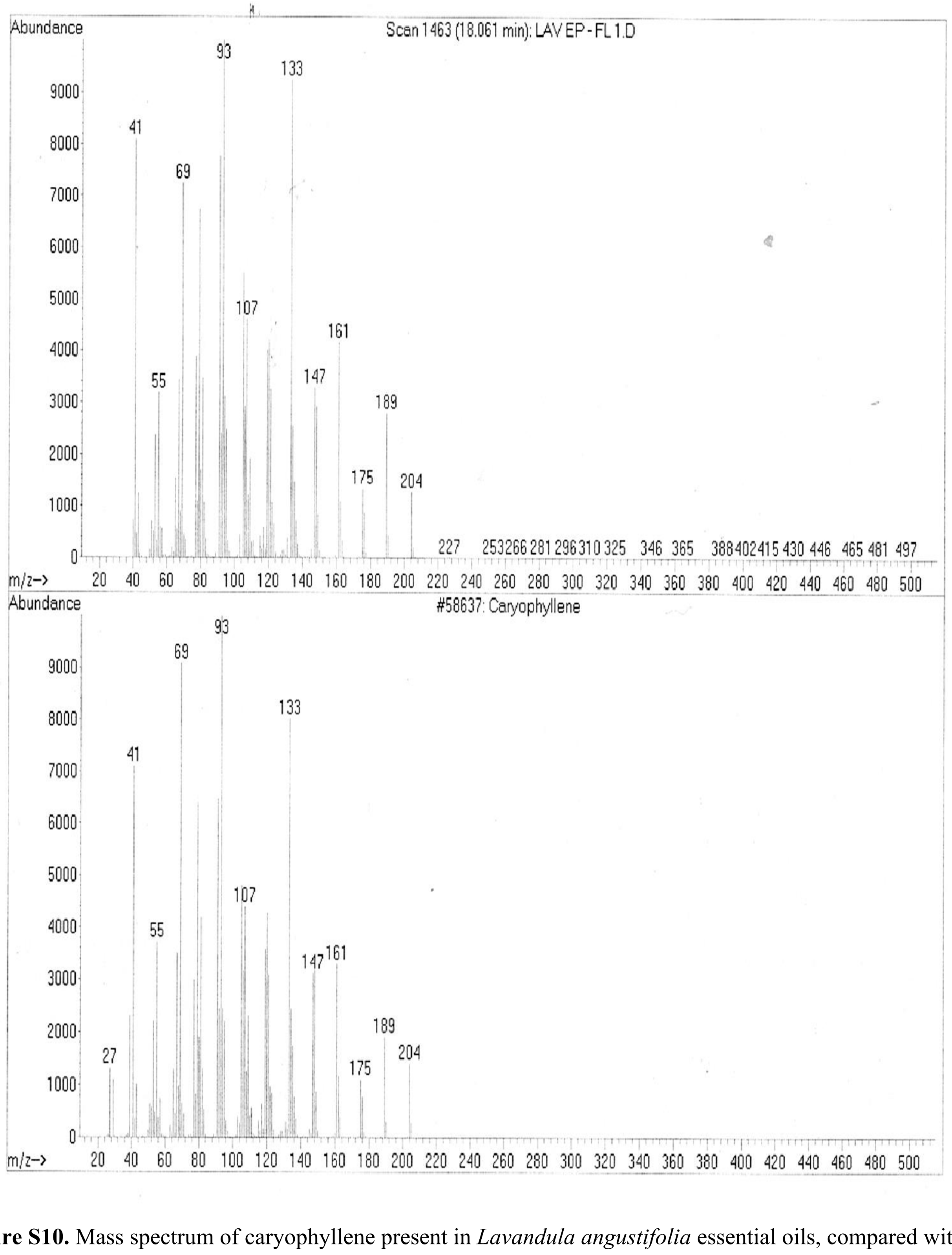

Figure S10. Mass spectrum of caryophyllene present in *Lavandula angustifolia* essential oils, compared with caryophyllene standard mass spectrum from NIST 02 library

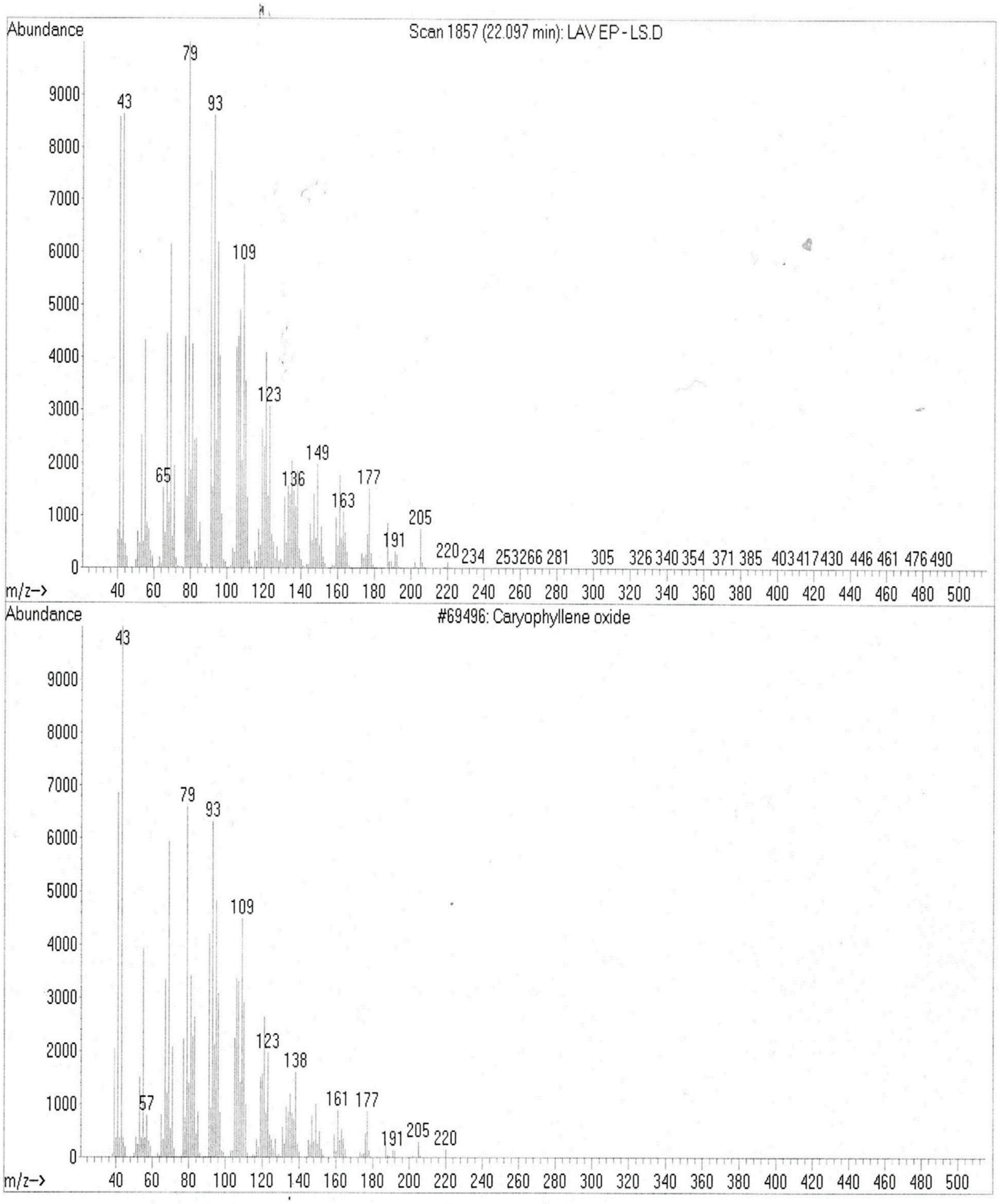

Figure S11. Mass spectrum of caryophyllene oxide present in *Lavandula angustifolia* essential oils, compared with caryophyllene oxide standard mass spectrum from NIST 02 library

PART III - GC-MS data of linalool standard

Figure S12. Chromatogram of linalool

Figure S13. Mass spectrum of linalool, compared with linalool standard mass spectrum from NIST 02 library

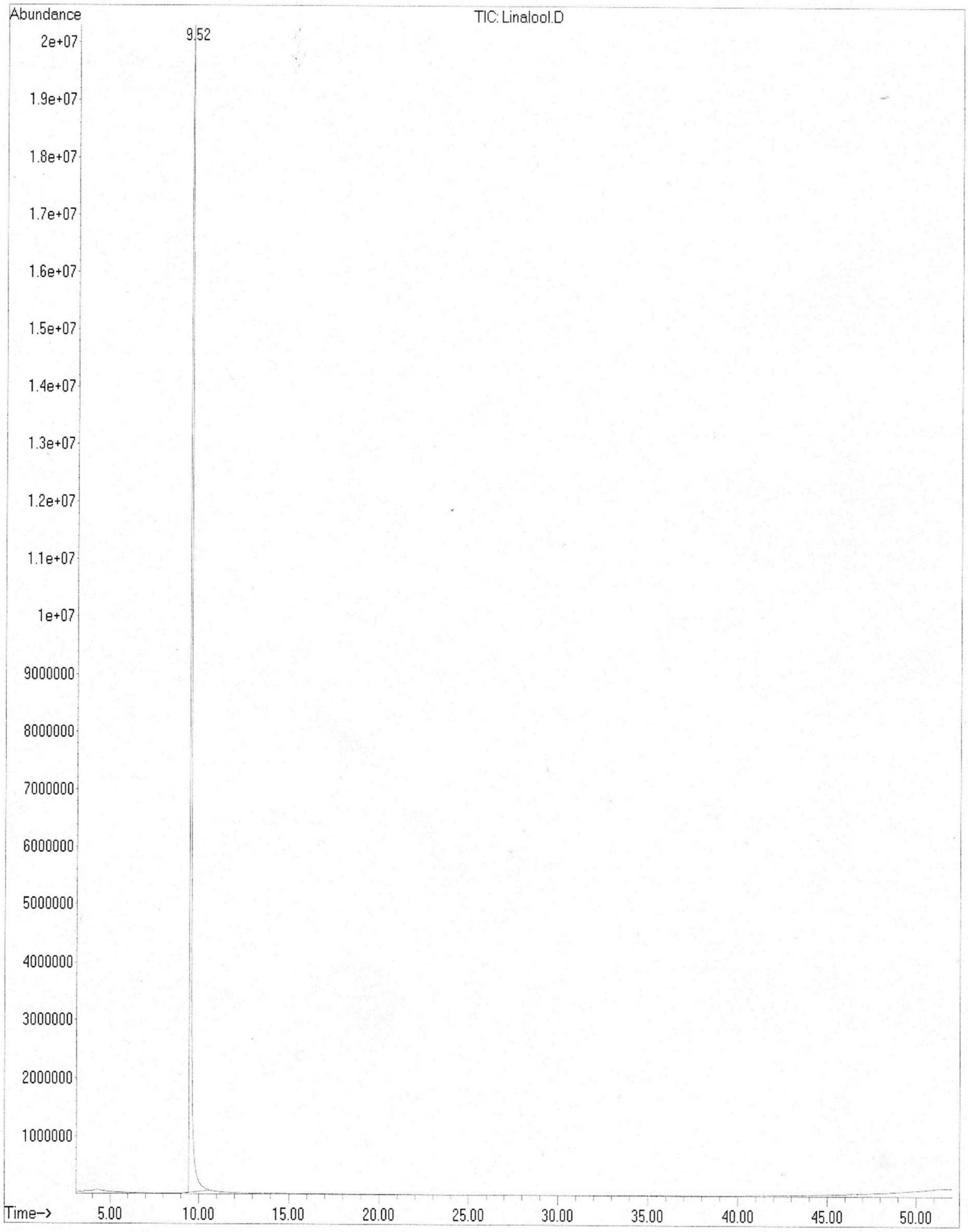

Figure S12. Chromatogram of linalool

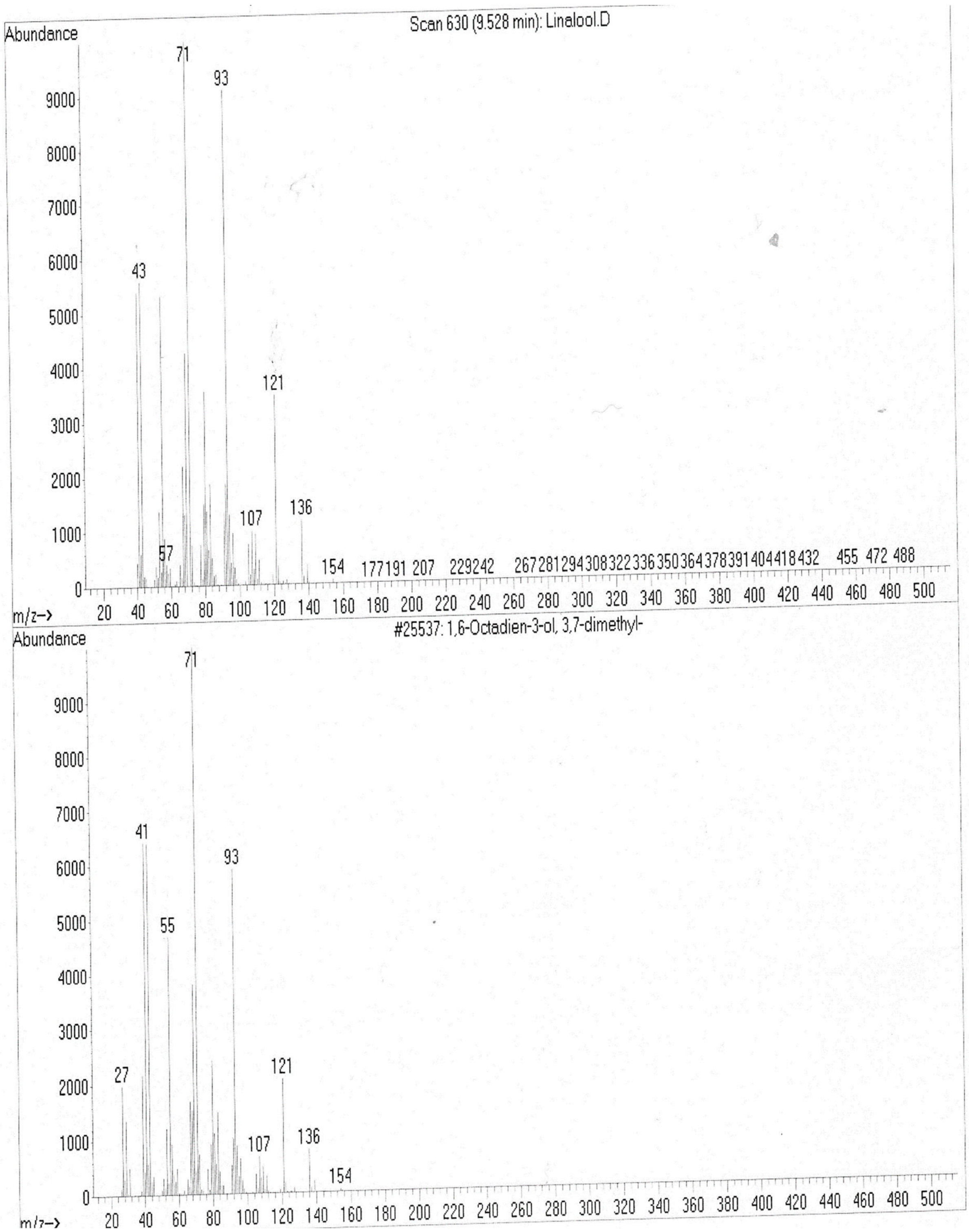

Figure S13. Mass spectrum of linalool, compared with linalool standard mass spectrum from NIST 02 library
